# Association Between Plasma Fibulin-1 and Brachial-Ankle Pulse Wave Velocity in Arterial Stiffness

**DOI:** 10.3389/fcvm.2022.837490

**Published:** 2022-07-07

**Authors:** Mandi Luo, Dan Yan, Xiaolu Liang, Yi Huang, Pengcheng Luo, Zhen Yang, Yucong Zhang, Ting Xu, Shangbang Gao, Le Zhang, Yiwu Zhou, Qing Shi, Cuntai Zhang, Lei Ruan

**Affiliations:** ^1^Department of Geriatrics, Tongji Hospital, Tongji Medical College, Huazhong University of Science and Technology, Wuhan, China; ^2^Laboratory of Molecular Biophysics of the Ministry of Education, College of Life Sciences and Technology, Huazhong University of Science and Technology, Wuhan, China; ^3^Department of Forensic Medicine, Tongji Medical College, Huazhong University of Science and Technology, Wuhan, China

**Keywords:** fibulin-1, pulse wave velocity, extracellular matrix, proteomics, arterial stiffness

## Abstract

Arterial stiffness forms the basis of cardiovascular diseases (CVD) and is also an independent predictor of CVD risk. Early detection and intervention of arterial stiffness are important for improving the global burden of CVD. Pulse wave velocity (PWV) is the gold standard for assessing arterial stiffness and the molecular mechanism of arterial stiffness remains to be studied. Extracellular matrix (ECM) remodeling is one of the major mechanisms of arterial stiffness. Partial quantitative changes of ECM proteins can be detected in plasma. Therefore, we examined the hypothesis that a discovery proteomic comparison of plasma proteins between high arterial stiffness (baPWV ≥ 1,400 cm/s) and normal arterial stiffness (baPWV < 1,400 cm/s) populations might identify relevant changed ECM proteins for arterial stiffness. Plasma samples were randomly selected from normal arterial stiffness (*n* = 6) and high arterial stiffness (*n* = 6) people. Isobaric tags for relative and absolute quantitation (iTRAQ) based quantitative proteomics technique was performed to find a total of 169 differentially expressed proteins (DEPs). Nine ECM proteins were included in all DEPs and were all up-regulated proteins. Fibulin-1 had the highest statistically fold-change (FC = 3.7, *p* < 0.0001) in the high arterial stiffness population compared with the control group during the nine ECM proteins. The expression of plasma fibulin-1 in normal arterial stiffness (*n* = 112) and high arterial stiffness (*n* = 72) populations was confirmed through enzyme-linked immunosorbent assay (ELISA). Similarly, ELISA results showed that plasma concentrations of fibulin-1 in the high arterial stiffness group were higher than those in the normal arterial stiffness group (12.69 ± 0.89 vs. 9.84 ± 0.71 μg/ml, *p* < 0.05). Univariate analysis of fibulin-1 with brachial-ankle pulse wave velocity (baPWV) indicated that fibulin-1 was positively correlated with baPWV in all participants (*r* = 0.32, *p* < 0.01) and a stronger positive correlation between baPWV and fibulin-1 in high arterial stiffness group (*r* = 0.64, *p* < 0.0001) was found. Multiple regression analysis of factors affecting baPWV showed that fibulin-1 was also a significant determinant of the increased ba-PWV (*R*^**2**^ = 0.635, *p* = 0.001). Partial correlation analysis showed that baPWV increased with the growth of plasma fibulin-1(*r* = 0.267, *p* < 0.001). In conclusion, our results demonstrated that fibulin-1 is positively correlated with ba-PWV and an independent risk factor for arterial stiffness.

## Introduction

Cardiovascular diseases (CVD) are the leading cause of death worldwide and arterial stiffness is considered an independent risk factor for it (1–3). Increased systolic blood pressure (SBP) due to arterial stiffness increases the risk of a range of CVD, such as hypertension, coronary heart disease, stroke, and arterial fibrillation (4–7). A multi-ethnic study of atherosclerosis (MESA) by Duprez and Jacobs et al. demonstrated that pulse wave velocity (PWV) is positively associated with incident congestive heart failure (CHF) in the general population (8). In addition, arterial stiffness is also one of the phenotypes of vascular aging (9). A growing body of evidence manifests that vascular aging can aggravate the risk of CVD (10). Epidemiological studies have shown that arterial hardness has a higher predictive value than traditional cardiovascular risk factors in determining cardiovascular risk and has become an important predictor of end-organ damage and overall risk of CVD (11–14). The inducement and molecular mechanism of arterial stiffness are very complex and unsolved. Therefore, the identification of potential molecules involved in arterial stiffness is of great significance to the early diagnosis and intervention of arterial stiffness, which is critical to the prevention of CVD.

At present, the assessment method of arterial stiffness is a mainly functional test and the most common method for arterial stiffness detection is pulse wave velocity (PWV) (15–17). PWV is a simple, effective, and economical non-invasive index including carotid-femoral pulse wave velocity (cfPWV), brachial-ankle pulse wave velocity (baPWV), and heart-femoral pulse wave velocity (hfPWV) and baPWV is more convenient and commonly used in the clinic (9, 17). Numerous studies have shown that PWV is elevated in arterial stiffness-related diseases such as atherosclerosis, diabetes, hypertension, and vascular aging (17–22). Small amounts of plasma proteins are associated with arterial stiffness due to disease (23–25). BaPWV≥ or <1,400cm/s was, respectively considered high arterial stiffness or normal arterial stiffness, which is the internationally current cut-off value for arterial stiffness currently (25–29).

Vascular remodeling is the major pathological characteristic of arterial stiffness, mainly manifested as extracellular matrix (ECM) remodeling and the transformation from contractile to the secretory phenotype of the vascular smooth muscle cell (VSMC) (7, 30, 31). ECM remodeling is a key step in the progression of arterial stiffness, including deposition of collagen, fragmentation of elastin in the medium layer, and cross-linking of collagen and elastin due to advanced glycation end products (AGE) (7, 32, 33). There may be some quantitative changes in ECM proteins during ECM rearrangement in arterial stiffness. While ECM proteins can be secreted into the blood, this quantitative change may be reflected in plasma (34).

Therefore, our study aimed to screen for ECM proteins that may be involved in quantitative changes in arterial stiffness by comparing plasma samples from people with normal (baPWV < 1,400 cm/s) or high arterial stiffness (baPWV ≥ 1,400 cm/s). We adopted isobaric tags for relative and absolute quantitation (iTRAQ) labeling followed by liquid chromatography-mass spectrometry (LC/MS) technology to study the differentially expressed proteins (DEPs) in plasma of people with normal or high arterial stiffness. Based on proteomic analysis results, fibulin-1, one of the differentially expressed ECM proteins, was screened out. We then preliminarily explored the relationship between plasma fibulin-1 and arterial stiffness.

## Materials and Methods

### Characteristics of the Enrolled Subjects

In this study, blood samples and clinical data were collected from individuals who underwent a regular physical examination at the Outpatient Physical Examination Center of Tongji Hospital, Tongji Medical College, Huazhong University of Science and Technology from January 2018 to December 2020. The ethic of our research was permitted by the Ethics Committee of Tongji Hospital, Huazhong University of Science and Technology (TJ-IRB20191215). The basic clinical data covered gender, age, height, weight, BMI (body mass index), baPWV, AI (arteriosclerosis index), SBP, DBP (diastolic blood pressure), PP (pulse pressure), MAP (mean arterial pressure), FBG (fasting blood glucose), TC (total cholesterol), TG (triglyceride), HDL-C (high-density lipoprotein cholesterol), LDL-C (low-density lipoprotein cholesterol), type 2 diabetes, hyperlipidemia, and medication status. The above clinical test indicators were all included in the health examination package. Exclusion criteria for the study population included: age < 18 and age > 90, individuals with cardiovascular diseases, autoimmune diseases, tumors, hematological diseases, thyroid diseases, severe liver and kidney dysfunction, acute or chronic infections, medication history, and incomplete basic case data. In the early stage of the study, physical examinations collected from January 2018 to July 2018 were screened according to the exclusion criteria mentioned above. Finally, 12 cases aged 20–70 years (6 cases of high arterial stiffness and six cases of normal arterial stiffness) were included for plasma proteomics testing to screen plasmic markers for arterial stiffness (Population 1). In the late stage of the study, physical examination data collected from January 2018 to December 2020 were screened according to the exclusion criteria adjusted for sex. Finally, 184 cases aged 19–84 years (72 cases of high arterial stiffness and 112 cases of normal arterial stiffness) were included for enzyme-linked immunosorbent assay (ELISA) (Population 2). Detailed characteristics of the above two populations have been demonstrated in Tables 1, 2. Data from participants of all groups were applied for statistical analysis.

**Table 1:** Clinical and demographic characteristics of the proteomic study population.

	**Normal arterial stiffness**	**High arterial stiffness**	***P*-value**
	**(*n* = 6)**	**(*n* = 6)**	
baPWV (cm/s)^a^	1,074 ± 58.57	2,473 ± 838.9	<0.01
baPWV (cm/s)^b^	1,171 ± 147.7	2,376 ± 147.7	<0.05
AI	2.09 ± 0.62	2.82 ± 1.12	0.195
Gender [*n* (%)]			
Male	2 (2)	2 (2)	1.000
Female	4 (4)	4 (4)	
Age (year)	36.00 ± 12.23	54.00 ± 9.74	<0.05
Height (cm)	170.0 ± 7.69	165.7 ± 10.61	0.437
Weight (kg)	63.45 ± 7.44	70.58 ± 15.43	0.331
BMI	21.92 ± 1.84	25.57 ± 4.28	0.084
SBP (mmHg)	109.0 ± 4.15	158.3 ± 18.96	<0.01
DBP (mmHg)	62.45 ± 5.81	94.93 ± 19.31	<0.05
PP (mmHg)	46.55 ± 6.43	63.40 ± 9.12	<0.05
MAP (mmHg)	77.97 ± 4.37	116.1 ± 18.72	<0.05
FBG (mmol/L)	4.84 ± 0.32	6.30 ± 1.53	<0.05
TC (mmol/L)	4.04 ± 0.70	4.88 ± 0.69	0.062
TG (mmol/L)	0.89 ± 0.27	1.48 ± 0.46	<0.05
HDL-C (mmol/L)	1.37 ± 0.47	1.35 ± 0.34	0.929
LDL-C (mmol/L)	2.91 ± 0.89	3.12 ± 0.92	0.694
Hyperlipidemia [n (%)]			
Yes	0 (2)	4 (2)	<0.05
No	6 (4)	2 (4)	
Type 2 Diabetes [*n* (%)]			
Yes	0 (1)	2 (1)	0.121
No	6 (5)	4 (5)	

*baPWV, brachial-ankle pulse wave velocity; AI, Arteriosclerosis index; BMI, body mass index; SBP, systolic blood pressure; DBP, diastolic blood pressure; PP, pulse pressure; MAP, mean arterial pressure; TC, total cholesterol; TG, triglyceride; HDL-C, high-density lipoprotein cholesterol; LDL-C, low-density lipoprotein cholesterol; FBG, fasting blood glucose. Data were present as means ± SD or median (quartile 1, quartile 3) for normally or non-normally distributed continuous variables*.

a *Unadjusted mean value of baPWV for the non-paired t-test*.

b *Adjusted mean value of baPWV for analysis of covariance (ANCOVA) (adjusting for age, DBP, FBG, and hyperlipidemia). P-value is significant*.

**Table 2:** Clinical and demographic characteristics of ELISA assay population for fibulin-1.

	**Normal arterial stiffness**	**High arterial stiffness**	***P*-value**
	**(*n* = 112)**	**(*n* = 72)**	
baPWV (cm/s)^a^	1,193 ± 119.7	1,811 ± 367.0	<0.001
baPWV (cm/s)^b^	1,285 ± 21.97	1,668 ± 29.37	<0.001
Arteriosclerosis Index (AI)	2.57 ± 0.98	3.19 ± 1.15	<0.001
Fibulin-1 (ug/ml)^a^	10.05 ± 6.66	12.37 ± 8.44	0.040
Fibulin-1 (ug/ml)^b^	9.84 ± 0.71	12.69 ± 0.89	0.016
Gender [*n* (%)]			
Male	68 (68.8)	45 (44.2)	0.877
Female	44 (43.2)	27 (27.8)	
Age (year)	46.33 ± 10.68	52.15 ± 11.55	0.001
Height (cm)	165.7 ± 16.02	165.6 ± 7.07	0.952
Weight (kg)	68.10 ± 18.25	67.13 ± 9.99	0.680
BMI (kg/m^2^)	24.16 ± 5.06	24.41 ± 2.88	0.699
SBP (mmHg)	114.3 ± 11.65	141.3 ± 17.90	<0.001
DBP (mmHg)	68.48 ± 10.51	85.94 ± 10.71	<0.001
PP (mmHg)	45.83 ± 7.87	55.40 ± 13.02	<0.001
MAP (mmHg)	83.76 ± 10.26	104.4 ± 12.07	<0.001
FBG (mmol/L)	4.86 ± 0.90	5.51 ± 1.45	<0.001
TC (mmol/L)	4.50 ± 0.86	4.94 ± 0.95	0.001
TG (mmol/L)	1.19 ± 0.66	1.72 ± 1.11	<0.001
HDL-C (mmol/L)	1.32 ± 0.34	1.23 ± 0.27	0.049
LDL-C (mmol/L)	2.79 ± 0.75	3.17 ± 0.87	0.002
Type 2 Diabetes [*n* (%)]			
Yes	3 (7.9)	10 (5.1)	0.006
No	109 (104.1)	62 (66.9)	
Hyperlipidemia [*n* (%)]			
Yes	39 (49.9)	43 (32.1)	0.001
No	73 (62.1)	29 (39.9)	

*baPWV, brachial-ankle pulse wave velocity; AI, Arteriosclerosis index; BMI, body mass index; SBP, systolic blood pressure; DBP, diastolic blood pressure; PP, pulse pressure; MAP, mean arterial pressure; TC, total cholesterol; TG, triglyceride; HDL-C, high-density lipoprotein cholesterol; and LDL-C, low-density lipoprotein cholesterol; FBG, fasting blood glucose. Data were present as means ± SD or median (quartile 1, quartile 3) for normally or non-normally distributed continuous variables and as frequency or percentage for categorical variables. The unpaired Student's t-test or the Wilcoxon signed-rank test was applied to evaluate statistical significance for continuous variables with or without normal distribution, respectively. Meanwhile, the Chi-square test was used to evaluate the statistics*.

a *Unadjusted mean value of fibulin-1or baPWV for the nonpaired t-test*.

b *Adjusted mean value of fibulin-1or baPWV for analysis of covariance (ANCOVA) (adjusting for age, SBP, DBP, MAP, PP, FBG, TG, TC, HDL-C, LDL-C, Type 2 diabetes, and hyperlipidemia). P-value is significant*.

### Baseline Measurement

Outpatient doctors collect information such as demographic characteristics, disease, and medication history of the physical examinees by the means of uniformed standardized consultation. Height and weight were canonically measured by a computer-human scale (SK L06B) and BMI was computed as the weight in kilograms divided by the square of the height in meters. SBP, DBP, and HR were measured by fixing an OMRON sphygmomanometer (Omron, Japan) to the appropriate position on the subjects' right arm after the subjects resting for at least 5 min in the seated position with hands and elbows flat on the table in the morning quietly. The blood pressure (BP) value was calculated as the average of three measured values. The PP equals SBP minus DBP and the MAP was calculated as (DBP+1/3×PP). Venous blood samples of the physical examination subjects after fasting for at least 8 h were collected with ethylene diamine tetraacetic acid (EDTA) purple anticoagulant tube at 8:30–11:30 am. Some were immediately sent to the clinical laboratory of Tongji Hospital for detection of TG, TC, LDL-C, HDL-C, and FBG. The AI which is an index of risk for arteriosclerosis was calculated as (TC–HDL-C)/HDL-C (35). And a portion of blood samples was collected, treated, and frozen for proteomics and ELISA. The blood samples were centrifuged at 3,000 rpm for 20 min at 4 °C and the plasma was collected. All plasma samples were stored at −80 °C until measurements. The pre-analysis conditions (sampling time, fasting, tube type, centrifugation, storage, etc.,) of plasma samples from Populatuion 1 and Population 2 included in the study for comparison were consistent.

### Pulse Wave Velocity Measurement

BaPWV was measured using the Vascular Profiler BP-203RPEIII system (Omron, Japan). The examination room was maintained at room temperature (RT, 22–25°C). Trained technicians placed four pressure cuffs on the subjects among others, one on the upper part of each arm and one on each ankle. Then, the subjects were examined after 15 min of rest in the supine position. The device simultaneously recorded the bilateral pulse waves of the brachial and posterior tibial arteries using an oscillometric method by extremities cuffs connected to a plethysmographic sensor and an oscillometric pressure sensor wrapped on both arms and ankles. The baPWV was calculated as the ratio of the traveled distance (which was automatically estimated from the body height) divided by the transit time of the pulse wave between the brachial and posterior tibial arteries. The time delay between the foot waveform of the ankle and brachial arteries is the transit time. The time delay between brachial and post-tibial arteries (Tba) was the transit time used for calculation. The wave conduction distances used to calculate baPWV included the distance from the suprasternal notch to the brachial artery (Dhb), from the suprasternal notch to the femur (Dhf), and from the femur to the ankle (Dfa). They were calculated automatically using the following equation (36–39):


(1) Dhb=(0.220×height {cm}−2.07)

(2) Dhf=(0.564×height {cm}−18.4)

(3) Dfa=(0.294×height {cm}+ 30.7)

BaPWV was calculated by the following equations:


(4) BaPWV=(Dhf+Dfa−Dhb)/Tba

The averages of the right-side and left-side baPWV values were used for the analysis. BaPWV < 1,400 cm/s was defined as normal arterial stiffness, and baPWV ≥ 1,400 cm/s was defined as high arterial stiffness (25–29).

### LC/MS-MS Analysis

Each blood sample from population 1 was taken appropriate amount for the extraction experiment. A protein lysate (7M Urea/2M Thiourea/4% SDS/40mM Tris-HCL, pH 8.5/1mm PMSF/2mM EDTA) was added to the sample, which was mixed and incubated on ice for 5 min. Then the sample was added with a final concentration of 10 mM DTT with ultrasound performed in an ice bath for 15 min. Next, the sample was centrifuged at 13,000 g at 4 °C for 20 min and the supernatant was transferred to a new centrifugal tube. Four times the volume of cold acetone was added to the centrifugal tube and left to stand overnight at −20 °C. Protein precipitates were collected by centrifugation and dried in air. Eight M Urea/100 mM TEAB (pH 8.0) solution was added to redissolve the protein and DTT were added to the protein to the final concentration of 10 mM to carry out a reduction reaction in a 56 °C water bath for 30 min. Next, IAM was added to the protein to a final concentration of 55 mm, and the alkylation reaction was carried out in the dark for 30 min at room temperature (RT, 22–25°C). Protein concentration was determined by the Bradford method (Sigma). One hundred microgrammes of protein mixture for each sample were used for trypsin digestion. The protein solution was diluted 5 times with 100 mM TEAB and added with trypsin in a mass ratio of 1:50 (trypsin: protein) to be placed at 37 °C overnight for enzymatic hydrolysis. After enzymatic hydrolysis, the peptides were desalted with a C18 column and freeze-dried in a vacuum. The peptides were dissolved with 0.5 M TEAB and labeled according to the iTRAQ Reagent8 plex standard kit (SCIEX) instruction. All of the labeled samples were mixed with an equal amount. Next, the labeled samples were fractionated using high-performance liquid chromatography (HPLC) system (Thermo DINOEX Ultimate 3,000 BioRS) using a Welch C18 (5 μm, 100 A, 4.6×250 mm) at high pH conditions. The separation of peptides was achieved by increasing the concentration of ACN under alkaline conditions at a flow rate of 1 mL/min, and one tube was collected per minute. A total of 42 secondary fractions were collected and combined into 15 components, which were desalted and vacuum dried on a STRATa-X column. The mass spectrometry data of the current study were collected using TripleTOF 5,600 + LC-MS/MS system (SCIEX). Peptide samples were dissolved in 2% acetonitrile/0.1% formic acid and analyzed using a TripleTOF 5600 PLUS mass spectrometer coupled to the Eksigent nanoLC system (SCIEX, USA). Polypeptide solution was added to a C18 capture column (5 μm, 100 μm×20 mm) and eluted on a C18 analysis column (3 μm, 75 μm×150 mm) at a time gradient of 90 min at a flow rate of 300 nL/min. The two mobile phases are buffer A (2% acetonitrile/0.1% formic acid/98% H_2_O) and buffer B (98% acetonitrile/0.1% formic acid/2% H_2_O). For information-dependent collection (IDA), the first-order mass spectrometry was scanned at 250 ms ion accumulation time whereas the second-order mass spectrometry of 30 precursor ions was collected at 50 ms ion accumulation time. The spectrum of MS1 was collected at the range of 350 to 1,500 m/z whereas that of MS2 was collected in the range of 100 to 1,500 m/z. The dynamic exclusion time of precursor ions was set to 15s.

### Proteome Identification and Quantification

The wiff format raw files generated by LC-MS/MS were subjected to ProteinpilotTM software (version 4.5; https://sciex.com.cn/) for proteome identification and quantification, using UniProt human sequence as the reference database. The protein pilot identification results were further filtered. The unused score of identified protein was considered to be greater than or equal to 1.3 (confidence level above 95%; *P* < 0.05). Further, the proteins containing at least one unique peptide for each protein were considered trusted proteins. The mean of the pairwise comparison ratio between repeated samples was used as the fold-changes of the samples to be compared. And the *p*-value of the Student's *T*-test of a single sample for pair-to-pair comparison between repeated samples was used as a *p*-value of significance difference test between samples to be compared. DEPs are screened according to fold-changes and *p*-value. Proteins with fold-changes ≥ 1.5 or ≤ 0.67 and *p*-values < 0.05 in at least one experiment replication were considered DEPs.

### Proteome Data Analysis

A volcano plot of all detected proteins was drawn using Graphpad Prism (version 9.2.0.332). Hierarchical cluster analysis of proteome data was also conducted using the heatmap package in the R language. A cluster of orthologous groups (COG) database (http://www.ncbi.nlm.nih.gov/COG) can also be used for orthologous protein classification. However, in the present study, the identified proteins were classified and grouped using the Kyoto Encyclopedia of Genes and Genomes (KEGG) database (http://www.genome.jp/kegg/).

### Enzyme-Linked Immunosorbent Assay

The levels of plasma fibulin-1 was analyzed using a sandwich enzyme immunoassay kit (MBL Research Product, CircuLeX, Japan). Plasma samples to be tested were first diluted with sample dilution buffer at a dilution ratio of 1:4,000. The standard and diluted samples to be tested were then added to the 96-well plate at the rate of 100 ul per well. The 96-well plate was sealed with plastic film and then incubated at room temperature (ca.25 °C) for 60 min with horizontal shaking at a speed of 300 rpm. After incubation, the 96-well plate was washed with 350 μl washing solution per well four times, and the liquid in the well was then drained off. The conjugated anti-human fibulin-1antibody [100 μl horseradish peroxidase (HRP)] was then added to each well and the 96-well plate was incubated at room ca.25 °C) for 60 min and shaken horizontally at a speed of 300 rpm. The 96-well plate was then cleaned with 350 μl washing solution per well four times and added with 100 ul substrate reagent each well incubated in dark at room temperature (ca.25 °C) for 10–20 min with shaken horizontally at a speed of 300 rpm. The reaction was stopped through the addition of 100 ul stop solution in each well. The absorbance value of each well was measured at 450nm. The standard curve was drawn according to the absorbance and concentration of the standard protein. Finally, the concentration of fibulin-1 in the sample was calculated according to the standard curve and the absorbance value of the test sample.

### Statistical Analysis

SPSS version 22.0 for O2Windows (SPSS Inc., USA) was applied to analyze the data obtained in the present study. Shapiro-Wilk test was performed to test the assumptions of normal distributions. Normally or non-normally distributed continuous variables are presented as the mean ± standard deviation (SD) or median (interquartile range). Categorical variables were presented as frequency or percentage. Differences between participants with high or normal arterial stiffness were compared using the *t*-test, Mann-Whitney U test, or Chi-squared test. Analysis of covariance (ANCOVA) was performed to assess the mean value of fibulin-1 or baPWV of two groups after adjusting for age, SBP, DBP, MAP, PP, FBG, TG, TC, HDL-C, LDL-C, Type 2 diabetes, and hyperlipidemia. Pearson's correlation was used to assess correlations between the parameters such as fibulin-1 and baPWV. Multiple linear regression stepwise analysis was used to assess the predictors of measures of arterial stiffness. Partial correlation analysis was used to analyze the changes of PWV with fibulin-1 after adjusting for age, SBP, DBP, MAP, PP, FBG, TG, TC, HDL-C, LDL-C, Type 2 diabetes, and hyperlipidemia (40). Two-tailed *P* values < 0.05 were considered statistically significant.

## Results

### Characteristics of the Proteomic Study Population

According to exclusion criteria and group standard, plasma of six normal arterial stiffness and 6 high arterial stiffness people were selected for the proteomic study. Results of the current study show that there was no significant difference in AI, gender distribution, height, weight, BMI, TC, HDL-C, LDL-C, and type 2 diabetes distribution between the study groups. Further, the high arterial stiffness population had significantly higher age, blood pressure, glucose, TG, and hyperlipidemia distribution as compared with the control group (Table 1). It was found that the unadjusted mean baPWV of the high arterial stiffness group was significantly higher than the normal arterial stiffness group (2,473 ± 838.9 vs. 1,074 ± 58.57 cm/s, *p* < 0.01). After adjusting for age, FBG, DBP, and hyperlipidemia that affect baPWV, the mean baPWV in the high arterial stiffness group still had a significantly higher mean baPWV than the normal arterial stiffness population (2,376 ± 147.7 vs. 1,171 ± 147.7 cm/s, *p* < 0.05).

### Identification of Differentially Expressed Proteins

Two batches of iTRAQ experiments were conducted in the present study. The protein numbers identified in batches 1 and 2 were 419 and 374, respectively. Further, it was noted that there was a total of 602 proteins (union) identified in the plasma of the high arterial stiffness group and normal arterial stiffness group matched in the UniProt Homo sapiens database, out of which 374 proteins (intersection) were present in both batches. DEPs referred to proteins whose fold-change (FC) was >1.5 or <0.67 with more than two peptides (*p* ≤ 0.05).

**Figure 1:**
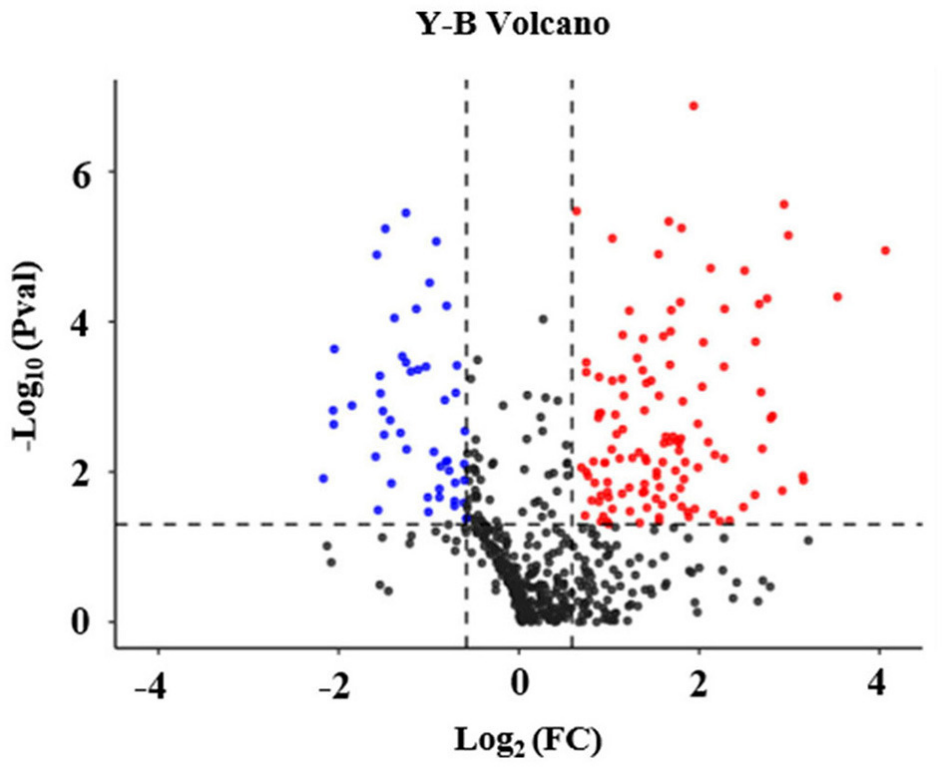
Volcano plot of differentially expressed proteins in plasma in the cheek-up crowd with normal or high arterial Stiffness. All proteins were plotted with log2 fold-change on the x-axis and – log10 (*P*-value) on the y-axis. The red dots in the upper right (ratio > 1.5) and the blue dots upper left (ratio <0.67) sections with *P* < 0.05 represent proteins that were significantly up and downregulated between the two groups. Black dots are proteins that were the same in the two compared groups.

**Figure 2:**
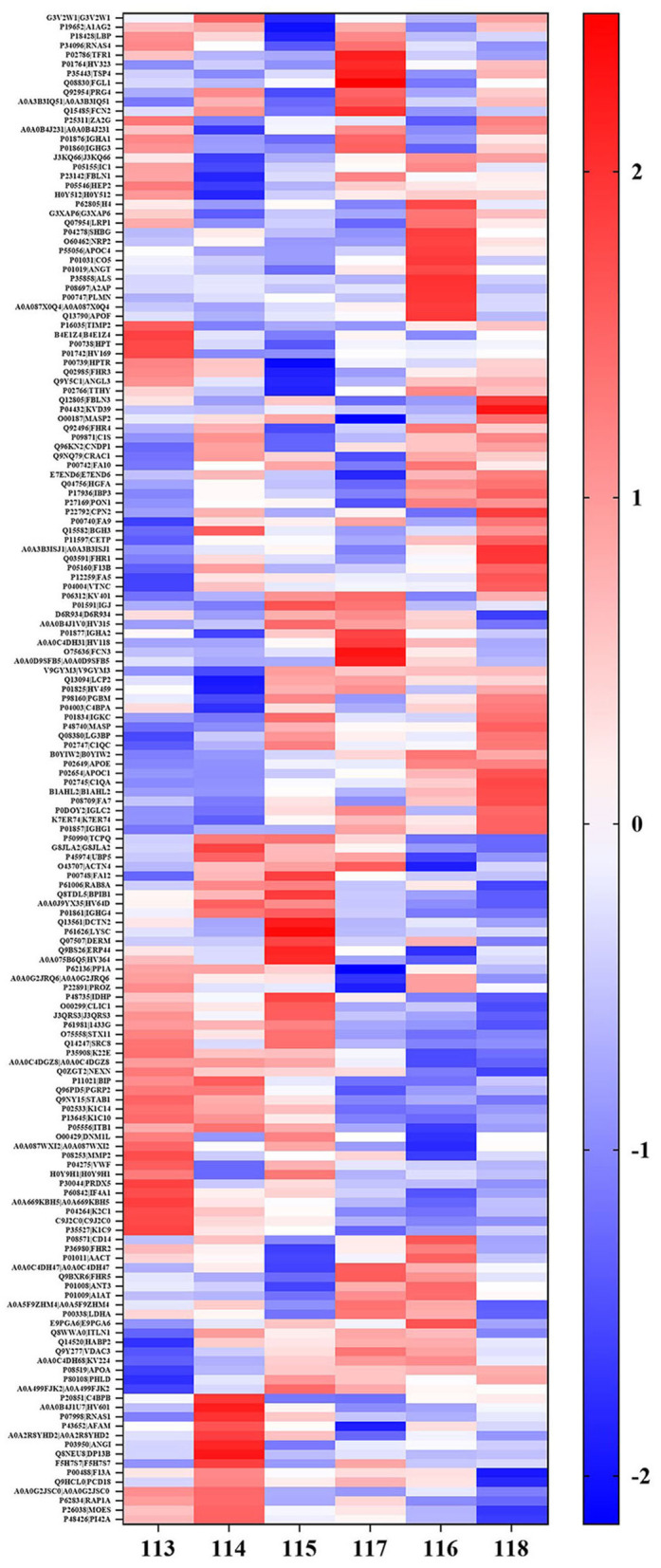
Hierarchical cluster analysis of the all detected proteins in plasma in cheek-up crowd with normal or high arterial Stiffness. Each column represents a plasma sample and each line represents a differentially expressed protein. Samples 113, 114, and 115 belong to normal arterial stiffness, and samples 116, 117, and 118 belong to high arterial stiffness. The color scale going from blue (low) to white (unchanged) and red (high) indicates the expression levels of differentially expressed proteins (DEPs). Red and blue indicate up-and-down-regulation, respectively whereas white indicates unchanged regulation.

In addition, it was found that out of the 602 identified proteins, 169 of them were significantly and DEPs were found in the high arterial stiffness group as compared with the controls whereby, 121 were upregulated and 48 were downregulated. Statistical analysis results of protein quantification were as presented in the volcano plot in Figure 1. The hierarchical clustering analysis displayed the abundance value of DEPs in each sample in plasma between the high arterial stiffness group and controls (Figure 2). Notably, fibulin-1, which is an ECM protein, was one of the upregulated proteins and up-regulated 3.7 times in the high arterial stiffness group as compared with the normal arterial stiffness group (Supplementary Table 1).

### Ontology and COG Annotation

**Figure 3:**
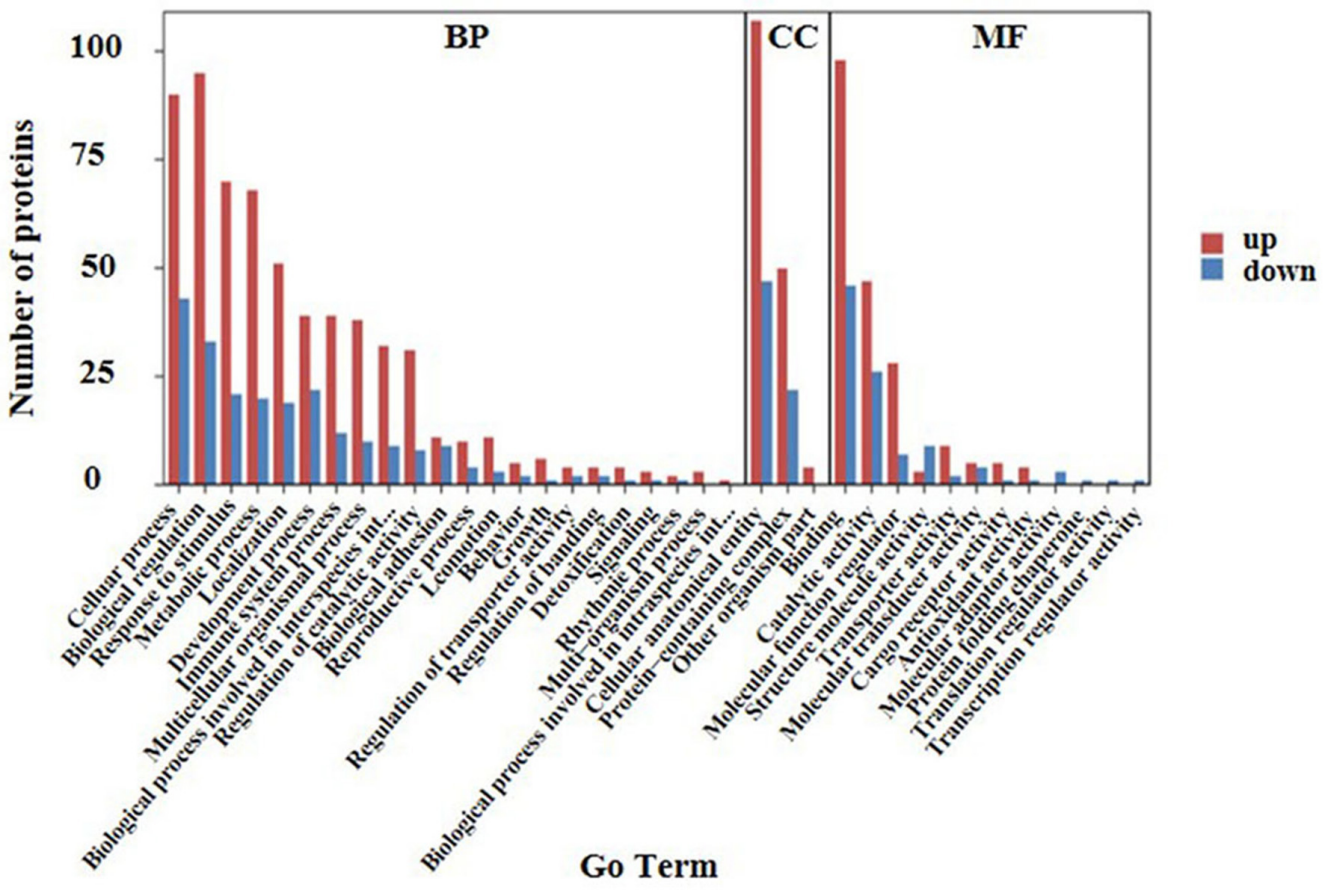
GO analysis of the 169 differential expressed proteins for functional classification. Red and blue bars represent up-regulation and down-regulation proteins. The BP, CC, and MF represent biological processes, cellular components, and molecular functions, respectively. Terms in the same category were ranked based on the number of proteins. The ordinates on the left represent the number of differentially expressed proteins (DEPs) in each entry.

Ontology (GO) functional annotation was used in the present study to uncover the functional classification of the 169 identified DEPs based on biological process (BP), molecular function (MF), and cellular component (CC) categories. Ontology enrichment analysis showed that the DEPs were mainly related to BP. Biological process analysis results in the current study showed that the majority of the proteins were primarily involved in cellular processes, biological regulation, response to stimulus, and localization. According to the results of the CC analysis, it was found that the DEPs were enriched in the cellular anatomical entity, protein-containing complex, and another organism part. Molecular function showed that the DEPs were primarily enriched in binding, catalytic activity, and molecular function regulation. In the above three items, up-regulated proteins were primarily involved in the cellular anatomical entity, biological regulation, cellular process, and binding (Figure 3).

**Figure 4:**
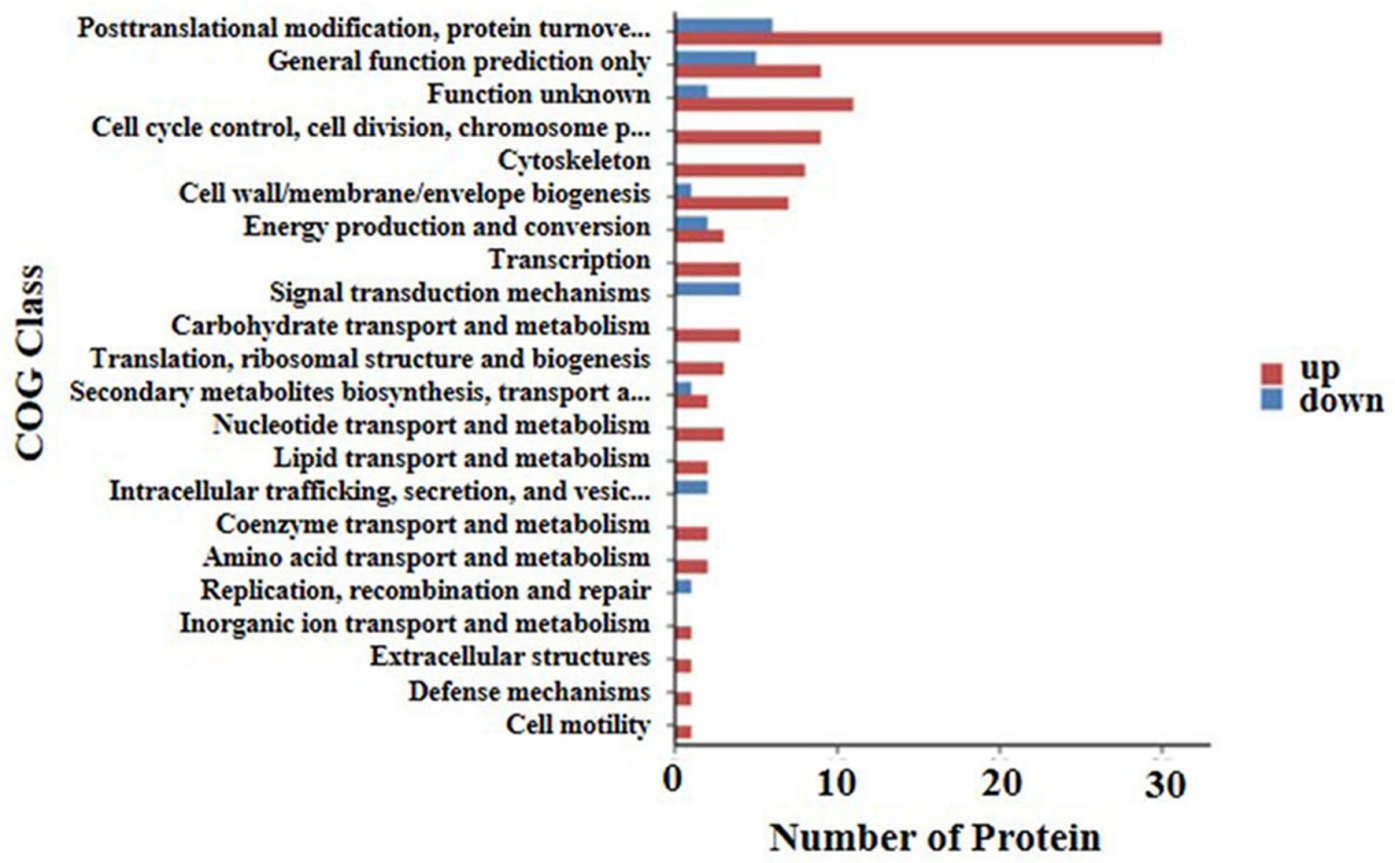
COG annotation of the 169 differential expressed proteins for functional classification. The y axis shows the cluster of orthologous groups of proteins class. The X-axis shows the number of detected proteins in each entry. The height of the red and blue bar represents the amount of detected up-regulated and down-regulated proteins of each item.

A cluster of orthologous groups (COG) annotation was used to compare the functional classifications of up-regulated and down-regulated proteins. Results of the COG annotation in the present study found that the function of the majority of DEPs was focused on posttranslational modification, protein turnover, chaperones, cell wall/membrane/envelope biogenesis, control of the cell cycle, cell division, chromosome partitioning, and signal transduction mechanisms. It was evident that there were a small number of proteins functioning in energy production and conversion as well as lipid transport and metabolism, extracellular structures, replication, recombination, and repair among others (Figure 4).

### Kyoto Encyclopedia of Genes and Genomes Pathway Enrichment Analysis

**Figure 5:**
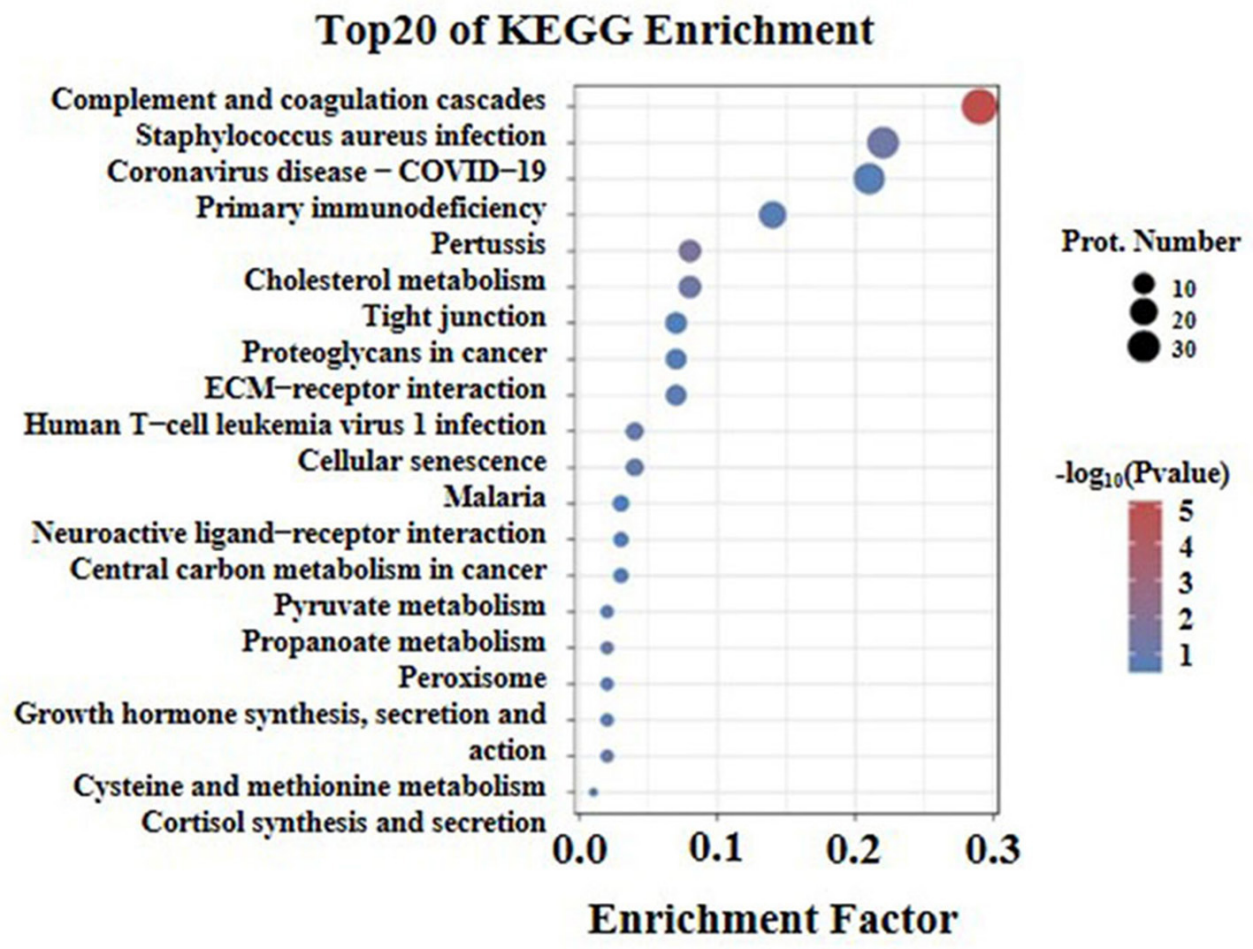
KEGG pathway enrichment analysis of differentially expressed proteins with the twenty highest enrichment scores. The x-axis shows the enrichment factor, the ratio of the number of differentially expressed proteins annotated with this pathway term to the total number of proteins annotated with this pathway term. A greater enrichment factor indicates greater intensiveness. The -log10 (*p*-value) ranges from 1 to 5 showing the enrichment score, the bigger the enrichment score, the smaller the *P*-value, indicating that the enrichment of differentially expressed proteins in given pathways was significantly different. The top 20 enriched pathway terms according to the KEGG database are shown; the *p*-value is <0.05.

The Kyoto Encyclopedia of Genes and Genomes pathway enrichment analysis (KEGG) was conducted to identify the functions of differentially expressed proteins. The results of the analysis showed that the first twenty KEGG pathways were enriched in the high arterial stiffness group compared with the control group. Further, it was found that the top ten significant pathways were as follows: complement and coagulation cascades, staphylococcus aureus infection, coronavirus disease-COVID-19, primary immune-deficiency, pertussis, cholesterol metabolism, tight junction, proteoglycans in cancer, ECM-receptor interaction, human T-cell leukemia virus 1 infection (Figure 5). The ECM receptor interaction pathway was ranked eighth among the ten significant pathways. Generally, the homeostasis of ECM in the vascular wall plays an important role in maintaining vascular structure and function, thus the imbalance of ECM will cause various vascular diseases. It is suggested that the difference in vascular hardness between the high arterial stiffness and normal arterial stiffness populations may be associated with the quantitative and qualitative changes in ECM.

### Characteristics of the ELISA Study Population

The characteristics of the ELISA assay population for fibulin-1 were as presented in Table 2. A total of 72 check-up crowds with high arterial stiffness and 112 check-up crowd with normal arterial stiffness were included in the present study for fibulin-1 according to exclusion criteria and adjusted for sex. No significant differences were found in sex, height, weight, and BMI between the investigated groups. Further, it was evident that the high arterial stiffness population was significantly different from the control subjects in terms of plasma fibulin-1, baPWV (1,811 ± 367.0 vs. 1,193 ± 119.7 cm/s, *p* < 0.001), blood pressure, blood lipids, type 2 diabetes, hyperlipidemia, age, and AI. The mean baPWV of high arterial stiffness was significantly higher than the population with normal arterial stiffness by adjusting for confounders including SBP, DBP, MAP, PP, FBG, TG, TC, HDL-C, LDL-C, Type 2 diabetes, and hyperlipidemia (1,668 ± 29.37 vs. 1,285 ± 21.97 cm/s, *p* < 0.001).

### Association Between BaPWV and Fibulin-1

**Figure 6:**
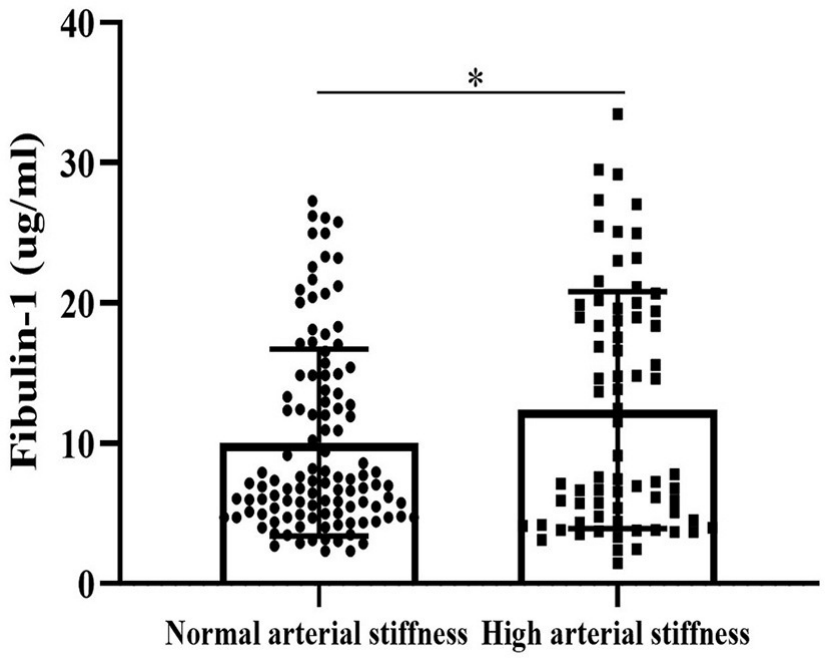
Fibulin-1 is upregulated in the arterial stiffness population. Plasma fibulin-1 levels were measured by an ELISA in people with normal arterial stiffness (*n* = 112) and high arterial stiffness (*n* = 72). Data are presented as columns (mean ± Standard Error of Mean).*P < 0.05 by Unpaired *t*-test.

**Figure 7:**
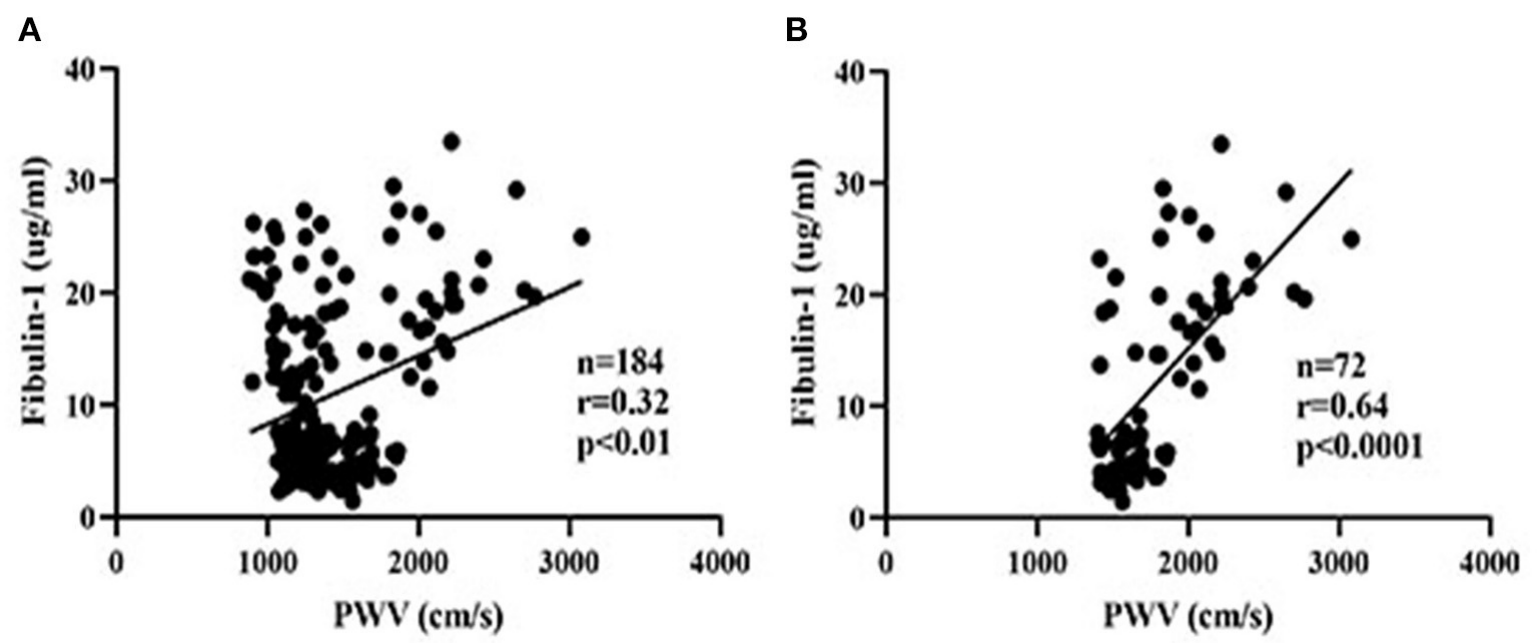
Scatter plots of plasma fibulin-1 and brachial-ankle pulse wave velocity. *r* = Pearson's correlation coefficient. **(A)** The relationship between plasma fibulin-1 and brachial-ankle pulse wave velocity in all study groups. **(B)** The relationship between plasma fibulin-1 and brachial-ankle pulse wave velocity in the high arterial stiffness group.

Results of our study indicated that the group with high arterial stiffness had significantly higher-level mean fibulin-1 in plasma as compared with the normal arterial stiffness group before adjusting for confounders (12.37 ± 8.44 vs. 10.05 ± 6.66 μg/ml, *p* < 0.05; Table 2, Figure 6). Interestingly, the signal variation of mean plasma fibulin-1 between the high arterialficant stiffness group and normal arterial stiffness group persisted after adjusting for age, SBP, DBP, MAP, PP, FBG, TG, TC, HDL-C, LDL-C, Type 2 diabetes, and hyperlipidemia (12.69 ± 0.89 vs. 9.84 ± 0.71 μg/ml, *p* < 0.05; Table 2). The simple linear correlation between arterial stiffness and study variables in their own included population were shown in Table 3. Further, it was found that there was a significant association between baPWV and fibulin-1. Results of univariate analysis in the current study indicated that fibulin-1 had a positive correlation with baPWV in all participants (*r* = 0.32, *p* < 0.01; Figure 7A). Furthermore, it was found there was a stronger positive correlation between baPWV and fibulin-1 in the high arterial stiffness group (*r* = 0.64, *p* < 0.0001; Figure 7B). Similarly, baPWV was also found to be positively associated with age, blood pressure, blood glucose, blood lipids, Type 2 diabetes, and hyperlipidemia (Table 3).

**Table 3:** Bivariate correlation analysis of baPWV in study participants about clinical and biochemic-al variables (*n* = 184).

**Variable**	**R**	***P*-value**
Fibulin-1 (ug/ml)	0.319	<0.001
Arteriosclerosis Index (AI)	0.280	<0.001
Age (year)	0.468	<0.001
Height (cm)	−0.007	0.461
Weight (kg)	0.006	0.468
BMI (kg/m^2^)	0.090	0.111
SBP (mmHg)	0.749	<0.001
DBP (mmHg)	0.646	<0.001
PP (mmHg)	0.523	<0.001
MAP (mmHg)	0.720	<0.001
FBG (mmol/L)	0.350	<0.001
TC (mmol/L)	0.202	0.003
TG (mmol/L)	0.242	<0.001
HDL (mmol/L)	−0.190	0.005
LDL (mmol/L)	0.222	0.001
Hyperlipidemia [*n* (%)]	0.207	0.002
Type 2 Diabetes [*n* (%)]	0.339	<0.001
Gender [*n* (%)]	0.086	0.124

*baPWV, brachial-ankle pulse wave velocity; AI, Arteriosclerosis index; BMI, body mass index; SBP, systolic blood pressure; DBP, diastolic blood pressure; PP, pulse pressure; MAP, mean arterial pressure; TC, total cholesterol; TG, triglyceride; HDL-C, high-density lipoprotein cholesterol; LDL-C, low-density lipoprotein cholesterol; FBG, fasting blood glucose. r is the Pearson index and P-value is its significance*.

**Figure 8:**
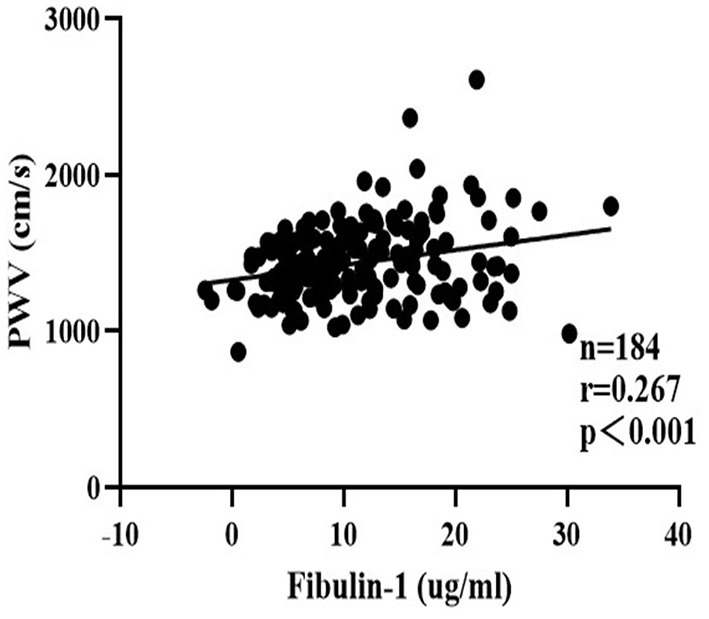
Scatter plots for partial correlation analysis of brachial-ankle pulse wave velocity and plasma fibulin-1. *r* = Partial correlation coefficient. Partial *r* and *p* values were obtained after adjustment for age, SBP, DBP, MAP, PP, FBG, TG, TC, HDL-C, LDL-C, Type 2 diabetes, and hyperlipidemia.

To eliminate the influence of confounding factors, a multiple regression analysis was performed in the present study to evaluate the effect of study variables in determining the baPWV. The baPWV was therefore used as the independent variable in multivariate analysis. Data entered into the models included sex, age, and other study variables significantly associated with baPWV including SBP, DBP, PP, MAP, TC, TG, HDL, LDL, FBP, Type 2 diabetes, hyperlipidemia, and fibulin-1 in the correlation analysis in Table 3. Results of the stepwise multiple regression analysis showed that age, blood pressure, and fibulin-1 were all associated with baPWV (Table 4). Fibulin-1, age, and blood pressure could explain 63.5% of the variation of the dependent variable baPWV (adjusted *R*^2^ = 0.635, *p* = 0.001) (Table 4). We can conclude that fibulin-1 is an important independent factor influencing the increase of baPWV in the study population in addition to age and blood pressure. Finally, to investigate whether baPWV changes with the concentration of plasma fibulin-1, a partial correlation analysis was performed by using baPWV as the dependent variable and fibulin-1 as the independent variable. Partial correlation analysis showed a weak positive linear correlation between baPWV and fibulin-1 and baPWV increased with the growth of plasma fibulin-1 (*r* = 0.267; *p* < 0.001; Figure 8).

**Table 4:** Predictors of measures of arterial stiffness (Results of multiple adjusted models).

**Dependent variables**	**Variables entered into models**	**Standardized β**	***R* ^2^**	***P***
BaPWV(cm/s)	Age (y)	8.642	0.635	0.001
	Fibulin-1(ug/ml)	8.077		
	SBP (mmHg)	12.68		

*baPWV, brachial-ankle pulse wave velocity; BMI, body mass index; SBP, systolic blood pressure; DBP, diastolic blood pressure; PP, pulse pressure; MAP, mean arterial pressure; TC, total cholesterol; TG, triglyceride; HDL-C, high-density lipoprotein cholesterol; LDL-C, low-density lipoprotein cholesterol; FBG, fasting blood glucose. Stepwise multiple linear regression analysis was performed. Standardized β provides a measure of the relative strength of the association independent of the measurement units. R^2^ is the goodness of fit of the multiple linear regression model. Covariates in the multiple-adjusted models included age, SBP, DBP, PP, MAP, TC, TG, HDL, LDL, FBP, Type 2 diabetes, hyperlipidemia, and plasma fibulin-1. P is the P-value of R^2^. Standardized β and P values were shown when P < 0.05*.

## Discussion

Our research found that plasma fibulin-1 is up-regulated in people with high arterial stiffness than the normal arterial stiffness group by proteomics in a small sample of people free of CVD. Then, plasma from a large sample of people free of CVD was used to find by ELISA that fibulin-1 was independently associated with baPWV and that the association was still present after adjusting for other risk factors for arterial stiffness. In addition, it has been found that baPWV increased with the growth of plasma fibulin-1 in a large sample of people free of CVD. These results are expected to provide a possible plasma marker for arterial stiffness and a new research idea for early detection and intervention of it, which has value in preventing cardiovascular adverse events (41).

The present study performed proteomic analysis of plasma proteins in both high arterial stiffness and normal arterial stiffness populations to reveal 169 DEPs, of which 121 were up-regulated proteins and 48 were down-regulated proteins. GOG analysis and KEGG analysis of these 169 DEPs showed that a small part of the 121 up-regulated proteins was involved in the formation of ECM structure. Furthermore, the most important feature of arterial stiffness is vascular remodeling, which is manifested by ECM remodeling in the media and phenotypic changes in VSMC (7, 30, 31). Therefore, the study focused on ECM proteins that are closely related to arterial stiffness among the up-regulated DEPs. Results of statistical analysis of the proteomic data revealed that nine ECM proteins were among the detected proteins with a fold-change >1.5. The ECM included fibulin-1, proteoglycan 4, metalloproteinase inhibitor 2, basement membrane-specific heparan sulfate proteoglycan core protein,72 kDa type IV collagenase, cartilage oligomeric matrix protein, lumican, and EGF-containing fibulin-like extracellular matrix protein (Supplementary Table 1). In addition, it was notable that fibulin-1 had the highest fold-change among the nine ECM proteins and thus we focused attention on the relationship between fibulin-1 and arterial stiffness.

Fibulin-1 belongs to one of the fibulins family composed of seven members and is an ECM and plasma glycoprotein that can interact with other ECM components, such as fibronectin, laminin, and fibrinogen, to stabilize the integrity of ECM (42, 43). Previous studies have shown that there are significant deposits of fibulin-1 in atherosclerotic lesions and thrombus, and fibulin-1 may thus play a role in the process related to the progression of atherosclerosis and thrombotic complications in humans (44). Elsewhere, Cangemi et al. (45) reported that the protein expression of fibulin-1 increased in the arterial wall of type 2 diabetes (T2D) and increased circulating concentration in patients with diabetes. In addition, it was found that both fibulin-1 and arterial stiffness index were increased in patients with diabetes (46). Further, it was evident that there was an independent association between serum fibulin-1 and augmentation index @75 (AIx@75), one of the important indicators to predict arterial stiffness, in the peripheral arterial disease group which suggested that fibulin-1 may play a vital role in arterial stiffness (47). In conclusion, fibulin-1 is closely related to arterial stiffness caused by diabetes mellitus, and atherosclerosis. However, the relationship between fibulin-1 and arterial stiffness independent of the disease remains unclear. We speculate that fibulin-1 may be involved in arterial stiffness in people with the non-specific disease. It was found in our research that the high arterial stiffness group (baPWV ≥ 1,400 cm/s) had a higher plasma concentration of fibulin-1 than those with normal arterial stiffness (baPWV < 1,400 cm/s) after adjusting for blood pressure, FBG, blood lipid, Type 2 diabetes, and hyperlipidemia. In addition, people with coronary heart disease, atherosclerosis, hyperuricemia, liver and kidney diseases, hypertension, and medication were eliminated to exclude the influence of diseases on the plasma concentration of fibulin-1 in our study. Therefore, our research suggested that there were more plasmic fibulin-1 in the high arterial stiffness population than in the vascular normal population in people without diseases. This result is consistent with the before-mentioned up-regulation of fibulin-1 in plasma of arterial stiffness-related diseases, further revealing a possible association between fibulin-1 and arterial stiffness (45–47). It was suggested that fibulin-1 may be associated with the occurrence of arterial stiffness independent of factors affecting arterial stiffness. Besides, the disease factor may promote arterial stiffness by causing the elevation of plasma fibulin-1.

Next, it was found that plasma fibulin-1 was positively correlated with baPWV through univariate linear regression. However, we also found that in addition to fibulin-1, age, blood pressure, and BMI were also correlated with baPWV. As expected, our results of this study showed that age, blood pressure, and fibulin-1 are all the main influencing factors of baPWV after multiple linear regression stepwise applied to screen risk factors for baPWV. A large number of studies have proved that age and blood pressure are the main influencing factors of baPWV (19, 48–50). Our results are consistent with existing studies on the relationship between baPWV and age and blood pressure, confirming the reliability of our sample data. In addition, fibulin-1 could still explain changes in baPWV after we adjusted the existing factors affecting baPWV in multiple linear regression, which indicates that fibulin-1 is indeed independently correlated with baPWV. This finding is consistent with the previous findings that fibulin-1 is associated with arterial stiffness due to disease (44–46, 51). Moreover, it is interesting to note that we found baPWV increased linearly with the increment of plasma fibulin-1 after concomitant variables were adjusted including age, SBP, DBP, MAP, PP, FBG, TG, TC, HDL-C, LDL-C, Type 2 diabetes and hyperlipidemia in partial correlation analysis. This result suggests that changes in plasma fibulin-1 independently affect changes in baPWV when other factors influencing baPWV are excluded, which is consistent with our previous results of multiple linear regression. Although our results suggested a bi-directional association between the increase of plasma fibulin-1 and baPWV, the causal relationship between the two needs to be explored further. This discovery we found provides new ideas and targets for early diagnosis and intervention of arterial stiffness and prevention of diseases caused by it.

However, there are some limitations to this study: (1) This project is a cross-sectional study with a small sample, and the analysis method is relatively simple, which can not prove the cause-effect relationship between fibulin-1 and arterial stiffness. It can only provide preliminary clues for exploring the association between them. Therefore, further a large-scale longitudinal study is needed to verify the changes in plasma concentration of fibulin-1 with different degrees of arterial stiffness. (2) Fibulin-1 was observed to be related to arterial stiffness, but the specific mechanism of its involvement in arterial stiffness is still unclear. It was impossible to obtain clinical aortic samples from high arterial stiffness people, and the expression levels of fibulin-1 in the vascular wall of high arterial stiffness and normal arterial stiffness people can only be verified by mouse level. The specific mechanism of fibulin-1 in arterial stiffness needs to be further studied in FBLN VSMC-specific knockout mice. (3) The study also screened out many other ECM proteins by proteomics, such as lumican, proteoglycan 4, metalloproteinase inhibitor 2, basement membrane-specific heparan sulfate proteoglycan core protein,72 kDa type IV collagenase, and cartilage oligomeric matrix protein. However, there is a need for further study of the relationship between other plasma markers and arterial stiffness and whether the correlation between arterial stiffness and a group of ECM proteins was stronger than that between arterial stiffness and individual ECM protein.

The present study found that a novel ECM protein, fibulin-1, is closely related to arterial stiffness suggesting that this protein could be a potential biomarker for the detection of arterial stiffness. Fibulin-1 could be helpful for the early detection of arterial stiffness and arterial stiffness-related diseases. It is expected to provide researchers with a new intervention target for the treatment of arterial stiffness. However, whether fibulin-1 is part of the pathological process of arterial stiffness was still unclear. There is more research to be done to clarify the relationship between fibulin-1 and arterial stiffness.

## Data Availability Statement

The original contributions presented in the study are included in the article/Supplementary Material. The proteomics datasets presented in this study can be found in iProX. The accession number(s) of the proteomics datasets can be found below: https://www.iprox.cn/, IPX0003850000. Further inquiries can be directed to the corresponding author/s.

## Ethics Statement

The study was reviewed and approved by the Ethics Committee of Tongji Hospital, Huazhong University of Science and Technology (TJ-IRB20191215). The patients/participants provided their written informed consent to participate in this study.

## Author Contributions

DY and XL: language help and writing assistance. QS, YH, PL, ZY, and LZ: proofreading. SG, YZha, TX, YZho, LR, and CZ: discussion. All authors contributed to the article and approved the submitted version.

## Funding

This work was supported by the National Key R&D Program of China (Grant Number: 2020YFC2008000; principal investigator: CZ), the Major Technology Innovation of Hubei Province (Grant Number: 2019ACA141; principal investigator: CZ), and the National Natural Science Foundation (Grant Number: 81901428; principal investigator: PL).

## Conflict of Interest

The authors declare that the research was conducted in the absence of any commercial or financial relationships that could be construed as a potential conflict of interest.

## Publisher's Note

All claims expressed in this article are solely those of the authors and do not necessarily represent those of their affiliated organizations, or those of the publisher, the editors and the reviewers. Any product that may be evaluated in this article, or claim that may be made by its manufacturer, is not guaranteed or endorsed by the publisher.

## Supplementary Material

Supplementary Table 1 Nine ECM proteins among all detected proteins with a fold change greater than 1.5.

Supplementary Table 2 A complete list of proteins and their regulation from the proteome results.
