# Supplementary material for: Association Between Plasma Fibulin-1 and Brachial-Ankle Pulse Wave Velocity in Arterial Stiffness
Source: Front Cardiovasc Med. 2022 Jul 7;9:837490. doi: 10.3389/fcvm.2022.837490 (PMC9302601; doi:10.3389/fcvm.2022.837490)
Supplement: Supplementary Table 1 — Nine ECM proteins among all detected proteins with a fold change greater than 1.5. [file Table_1.DOCX]

Supplementary Material

# Supplementary Table 1: Nine extracellular matrix proteins among the all detected proteins in the plasma proteomics with fold change greater than 1.5.

| Accession | Name | Diff_state | Y: B | PVal Y: B |
| --- | --- | --- | --- | --- |
| sp\|P23142\|FBLN1_HUMAN | Fibulin-1 OS=Homo sapiens OX=9606 GN=FBLN1 PE=1 SV=4 | up | 3.174793863 | 0.00000 |
| sp\|Q92954\|PRG4_HUMAN | Proteoglycan 4 OS=Homo sapiens OX=9606 GN=PRG4 PE=1 SV=3 | up | 2.602667362 | 0.000170448 |
| sp\|P16035\|TIMP2_HUMAN | Metalloproteinase inhibitor 2 OS=Homo sapiens OX=9606 GN=TIMP2 PE=1 SV=2 | up | 2.345539845 | 0.033453943 |
| sp\|P98160\|PGBM_HUMAN | Basement membrane-specific heparan sulfate proteoglycan core protein OS=Homo sapiens OX=9606 GN=HSPG2 PE=1 SV=4 | up | 1.979912225 | 0.018432279 |
| sp\|Q9NQ79\|CRAC1_HUMAN | Cartilage acidic protein 1 OS=Homo sapiens OX=9606 GN=CRTAC1 PE=1 SV=2 | up | 1.973456813 | 0.046131963 |
| sp\|P08253\|MMP2_HUMAN | 72 kDa type IV collagenase OS=Homo sapiens OX=9606 GN=MMP2 PE=1 SV=2 | up | 1.840882672 | 0.001914665 |
| sp\|Q9NQ79\|CRAC1_HUMAN | Cartilage oligomeric matrix protein OS=Homo sapiens OX=9606 GN=COMP PE=1 SV=1 | up | 1.679228617 | 0.000352523 |
| sp\|P51884\|LUM_HUMAN | Lumican OS=Homo sapiens OX=9606 GN=LUM PE=1 SV=2 | up | 1.671540062 | 0.3251946 |
| sp\|Q12805\|FBLN3_HUMAN | EGF-containing fibulin-like extracellular matrix protein 1 OS=Homo sapiens OX=9606 GN=EFEMP1 PE=1 SV=2 | up | 1.660059479 | 0.038080062 |
